# Preimplantation Genetic Testing of Spinocerebellar Ataxia Type 2—Robust Tools for Direct and Indirect Detection of the ATXN2 CAG Repeat Expansion

**DOI:** 10.3390/ijms27031546

**Published:** 2026-02-04

**Authors:** Nur Asherah, Mulias Lian, Arnold S. Tan, Riho Taguchi, Pengyian Chua, Shuling Liu, Caroline G. Lee, Samuel S. Chong

**Affiliations:** 1 Department of Paediatrics, Yong Loo Lin School of Medicine, National University of Singapore, Singapore 119228, Singapore; 2 Preimplantation Genetic Diagnosis Centre, Department of Obstetrics and Gynaecology, National University Hospital, Singapore 119074, Singapore; 3 KKIVF Centre, Reproductive Medicine, KK Women’s & Children’s Hospital, Singapore 229899, Singapore; 4 Department of Biochemistry, Yong Loo Lin School of Medicine, National University of Singapore, Singapore 117596, Singapore; 5 Duke-NUS Medical School, Singapore 169857, Singapore; 6 Department of Obstetrics and Gynaecology, Yong Loo Lin School of Medicine, National University of Singapore, Singapore 119228, Singapore; 7 Molecular Diagnosis Centre, Department of Laboratory Medicine, National University Hospital, Singapore 119074, Singapore

**Keywords:** spinocerebellar ataxia type 2, multi-microsatellite haplotyping, triplet-primed PCR, preimplantation genetic testing for monogenic disorder

## Abstract

Spinocerebellar ataxia type 2 (SCA2) is an autosomal dominant neurodegenerative disorder caused by a pathogenic CAG trinucleotide repeat expansion in the *ATXN2* gene. At-risk couples can embark on unaffected pregnancies through preimplantation genetic testing of monogenic disorders (PGT-M) of SCA2, which should involve accurate repeat expansion detection together with risk haplotype tracking using informative linked markers. Two couples underwent SCA2 PGT-M involving analysis of whole genome amplified embryonic trophectoderm cells by *ATXN2* (CAG)n triplet-primed PCR (TP-PCR) and linkage-based risk allele genotyping using customized markers. To simplify and expedite the identification of informative markers for future PGT-M cases, putative microsatellite markers closely linked to *ATXN2* were initially screened for polymorphism using a small set of anonymous DNA samples obtained from Coriell Cell Repository. Shortlisted markers with high polymorphism likelihood were then multiplexed in a single-tube reaction and genotyped on 190 anonymous DNA samples to determine their polymorphic information content. Across both SCA2 PGT-M clinical cases, the linked marker genotypes corroborated the TP-PCR results, allowing clear differentiation between unaffected and affected embryos. In both cases, transfer of an unaffected embryo led to a successful pregnancy and live birth of a healthy baby. In silico mining, filtering, and curation identified 287 microsatellites located within 1.65 Mb of either side of the *ATXN2* CAG repeat. Of these, eight upstream and nine downstream polymorphic markers were successfully co-amplified in a single-tube assay and demonstrated high overall heterozygosity in both Chinese and Caucasian populations. Conclusion: To ensure high diagnostic accuracy for PGT-M of SCA2, we developed a heptadecaplex microsatellite marker panel for haplotype-based linkage analysis to complement TP-PCR-based direct detection of the *ATXN2* CAG repeat. The panel can rapidly identify informative markers from virtually any couple, and it works equally well on MDA-amplified DNAs for embryonic haplotype analysis.

## 1. Introduction

Spinocerebellar ataxia type 2 (SCA2; OMIM 183090) is a progressive neurodegenerative disorder characterized by cerebellar ataxia, dysarthria, and progressive gait instability due to a CAG trinucleotide repeat expansion in the *ATXN2* gene, located on chromosome 12q24.12 [1,2,3]. SCA2 is one of the more common subtypes of SCAs worldwide. It belongs to a broader group of polyglutamine (polyQ) disorders, with normal alleles typically ranging from 15 to 32 repeats [4,5]. Pathogenic alleles are those with 33 or more repeats [6], with full penetrance generally observed beyond 34 repeats. Repeat sizes of 37 to 39 are most commonly associated with clinical symptoms, although alleles exceeding 200 repeats have also been documented [3]. Larger repeat sizes correlate with earlier onset due to anticipation, a phenomenon where the disease severity and onset worsen in successive generations [2,4].

Due to the severity of SCA2 and its heritable nature, PGT-M presents an opportunity for at-risk couples who choose to undergo in vitro fertilization (IVF) to prevent the transmission of this disorder. PGT-M allows for the detection of pathogenic alleles in in vitro fertilized embryos and enables the selection of unaffected embryos for implantation [7], thus avoiding the need for termination should the embryo be affected.

PGT-M of SCA2 can be performed either through direct detection of the *ATXN2* CAG repeat expansion or via haplotype analysis using linked microsatellite markers on DNA obtained from embryo biopsies, after whole genome amplification (WGA). Direct detection of the SCA2 pathogenic repeat is most commonly performed using PCR-based methods such as fluorescent fragment analysis and triplet-primed PCR (TP-PCR) [8,9]. However, these methods are not without limitations. Standard PCR often fails to amplify very large expansions due to polymerase slippage or inefficiencies in repetitive sequences [10]. Mao R. et al. (2002) highlighted the importance of testing for large CAG expansions in paediatric cases of SCA2 and SCA7, as expansions of more than 100 repeats were identified in children presenting with early-onset ataxia [11]. The authors noted that while standard PCR can detect normal-sized alleles, it often fails to detect large pathogenic expansions, particularly in WGA samples. This increases the risk of false negatives in direct-only testing approaches, where failure to amplify the expanded allele may lead to misdiagnosis [11].

TP-PCR improves detection sensitivity, especially for expanded alleles, and may be more suitable than standard PCR. TP-PCR overcomes the limitations of standard PCR with the use of a third primer that anneals at multiple positions within the CAG tract, ensuring that the repeat itself is amplified, reducing the risk of failed amplification of the expanded allele. Multiple studies have demonstrated that TP-PCR performs better than standard PCR when detecting trinucleotide repeat disorders [12,13,14]. However, TP-PCR is still susceptible to allele dropout (ADO), preferential amplification and other amplification biases [15,16]. These issues are problematic, especially in PGT-M, where the limited amount of embryonic DNA is often subjected to WGA, which can introduce further bias [17].

In PGT-M analysis, microsatellite markers are often used for indirect detection of pathogen-associated alleles through haplotype-based linkage analysis. These microsatellite markers flanking the disease-causing gene are genotyped in family members to establish disease-associated and normal haplotypes. The use of linkage analysis with microsatellite markers complements direct detection of the pathogenic variant in PGT-M and serves as a safeguard in situations where amplification of the pathogenic allele is unsuccessful.

Additionally, in the case of repeat expansion disorders where dropout of the pathogenic allele cannot be ruled out because both parents carry identically sized normal alleles, microsatellite-based haplotyping enables clearer interpretation of inheritance patterns, thereby allowing for more accurate embryo diagnosis.

Both direct testing of the *ATXN2* CAG repeat and indirect microsatellite-based haplotyping can be used for affected couples who opt for IVF-PGT-M for SCA2. Although either method can be used independently, combining both provides higher diagnostic confidence when used to detect the inheritance of the pathogenic allele in embryos. In this study, two clinical SCA2 PGT-M cases were evaluated using TP-PCR to detect the expanded allele, with linked microsatellite markers used for haplotype confirmation. Similar linkage-based strategies have been successfully applied in the PGT-M of other trinucleotide disorders such as spinocerebellar ataxia type 3 (SCA3) [18], Huntington disease [19], fragile-X syndrome [20], and myotonic dystrophy [21]. To facilitate the efficient identification of informative microsatellite markers for linkage analysis in couples seeking PGT-M for SCA2, microsatellite markers located within 1 Mb upstream to 1.65 Mb downstream of the *ATXN2* gene were identified and assessed on their polymorphic information content, amplification performance, and heterozygosity in a reference sample population.

## 2. Results

### 2.1. Clinical IVF-PGT-M for SCA2

In the first couple, the SCA2 TP-PCR protocol was optimized on 10 ng of parental genomic DNA and subsequently validated on the WGA product generated from GM04866 single cells. For the second couple, SCA2 TP-PCR validation was performed on the WGA product generated from 100 pg of genomic DNA of the affected spouse. The SCA2 TP-PCR assay involves three primers, namely a gene-specific flanking primer located upstream of the CAG repeat tract, an opposing triplet-primed (TP) primer composed of (from 3′ to 5′) five CTG repeats, three bases complementary to the three nucleotides immediately downstream of the repeat tract, and a 20-nucleotide non-human sequence, as well as a Tail primer whose sequence is identical of the TP primer’s 20-nucleotide non-human sequence. The non-human tail sequence does not anneal to the human genome but is meant to provide a common binding site on all amplified fragments for the Tail primer to anneal to, enabling further amplification of the TP-PCR products.

**Figure 1:**
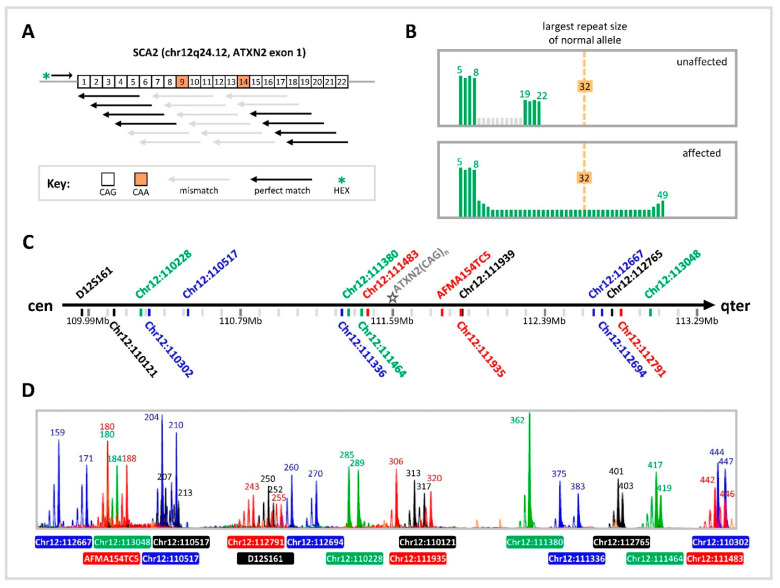
PGT-M of SCA2 by TP-PCR of the *ATXN2* CAG repeat and haplotype analysis of flanking microsatellite markers. (**A**) *ATXN2* CAG repeat structure and TP-PCR primer annealing positions, (**B**) expected TP-PCR electropherogram patterns of unaffected and affected individuals, (**C**) relative position of 17 polymorphic microsatellite markers within 1.65 Mb of either side of the *ATXN2* CAG repeat, and (**D**) representative electropherogram of heptadecaplex marker PCR products from 10 ng genomic DNA. Marker alleles represented by blue, green, red, and black peaks are tagged with 6-FAM, VIC, PET and NED fluorophores, respectively.

Since the TP primer consists of five CTG repeats and anneals to multiple positions within the CAG tract, the shortest TP-PCR product contains the first five uninterrupted CAGs at the 5′ end of the repeat tract, closest to the upstream gene-specific flanking primer. As a result, the leftmost TP-PCR peak in the electropherogram represents the first five CAG trinucleotides at the 5′ end of the *ATXN2* repeat tract. Successive TP-PCR peaks are longer by 3 bp, with the final, tallest peak corresponding to the longest fragment, allowing the repeat length to be determined by peak counting. However, the *ATXN2* CAG tract typically contains CAA interruptions at specific positions, commonly at the 9th and 14th repeats. The TP primer does not anneal efficiently across these interruptions, resulting in a gap in peak signal between the 8th and 15th repeat (Figure 1A,B). After the interruptions, the TP primer is again able to anneal efficiently, and strong peaks reappear after the gap in peak signal.

The unique TP primer sequence results in a terminal fluorescent peak that is significantly taller than the preceding few peaks. This design allows for easier identification of the largest/longest TP-PCR fragment, thereby enabling accurate determination of the CAG repeat length of the expanded allele in the sample by simply counting the number of fluorescent peaks starting from the shortest fragment containing five repeats. This occurs because the TP primer binds at multiple positions within the CAG repeat tract, generating a ladder of fragments of increasing length. When the TP primer anneals to the most distal five CAGs, primer annealing extends into the unique flanking sequence beyond the repeat tract, enabling stronger primer annealing, and hence more efficient extension and amplification yield of the longest fragment. This increased yield appears as a taller terminal peak in the electropherogram.

**Figure 2:**
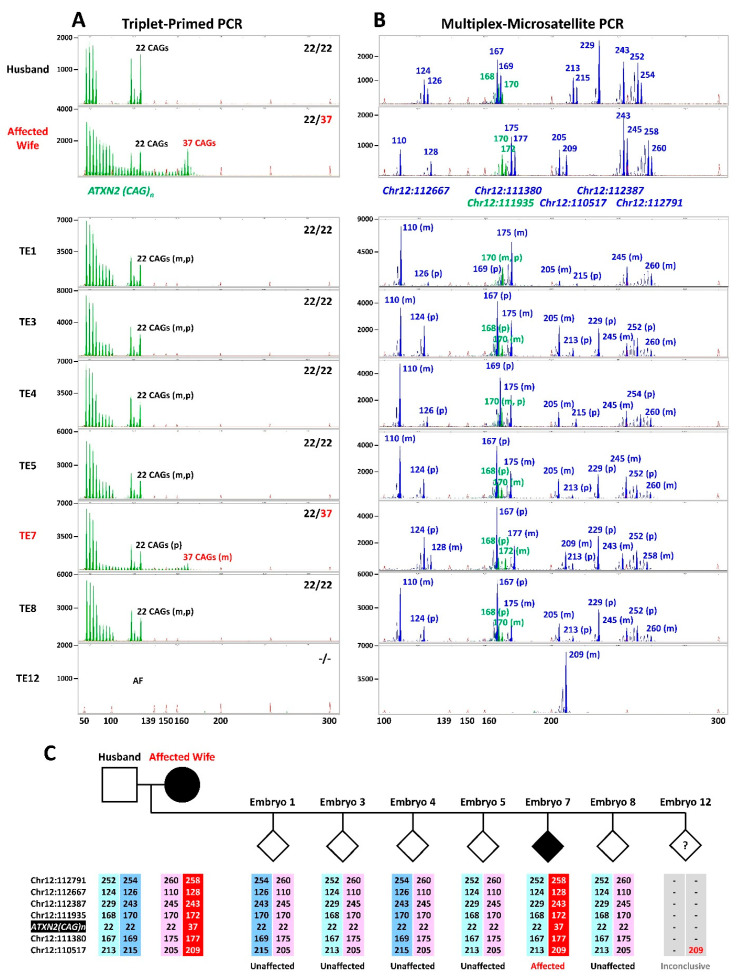
SCA2 clinical IVF-PGT-M case #1. (**A**) Electropherograms of triplet-primed PCR of the *ATXN2* CAG repeat. (**B**) Electropherograms of multiplex PCR of six linked microsatellite markers flanking the *ATXN2* CAG repeat, performed on 10 ng parental genomic DNAs and whole genome amplified trophectoderm biopsy samples. (**C**) Inherited paternal and maternal marker haplotypes of embryos. The maternal high-risk haplotype associated with the expanded allele is highlighted in red. Unaffected embryos are represented by empty diamond symbols, the affected embryo by a filled diamond symbol, and the embryo with an inconclusive diagnosis by a diamond symbol containing a question mark. TE, trophectoderm; AF, amplification failure; m, maternal allele; p, paternal allele.

The clinical cases of SCA2 employed a validated TP-PCR assay for direct sizing of *ATXN2* CAG repeats. To complement the common direct assay, linkage analysis was performed in parallel using marker panels customized for each couple. In the first clinical IVF-PGT-M case, the unaffected husband carries two normal 22-repeat *ATXN2* alleles, while the affected wife carries one normal 22-repeat allele and one expanded 37-repeat allele (Figure 2). Of seven embryos analyzed, TP-PCR results indicated that five embryos inherited the maternal normal allele and were diagnosed as unaffected, while one embryo inherited the maternal expanded allele and was diagnosed as affected. The remaining embryo did not yield successful amplification and could not be genotyped. Multiplex-marker analysis corroborated the TP-PCR results, with the five unaffected embryos inheriting the same maternal haplotype, which was therefore assigned as the low-risk haplotype. As expected, the affected embryo inherited the other (i.e., high-risk) maternal haplotype. The unamplified embryo displayed only a single allele of size 209 bp for marker Chr12:110517, which is present on the maternal high-risk haplotype. However, due to amplification failure of the remaining markers and the TP-PCR assay, no definitive diagnosis was possible. Transfer of one of the frozen–thawed unaffected embryos led to a successful pregnancy and the live birth of a healthy infant.

**Figure 3:**
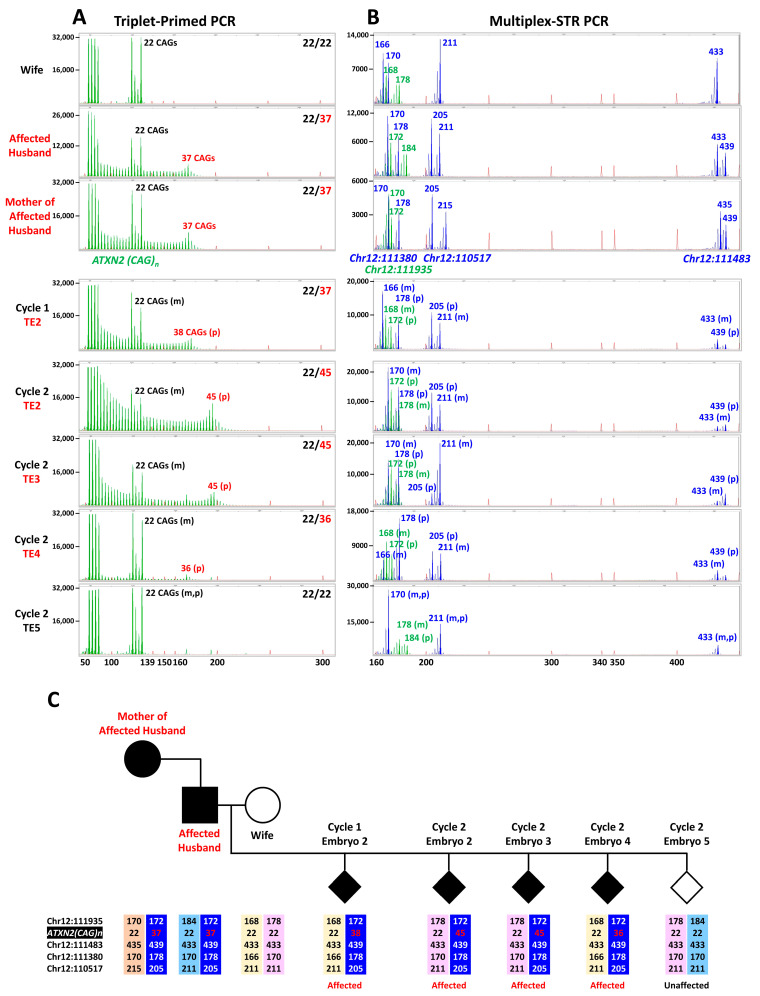
SCA2 clinical IVF-PGT-M case #2. (**A**) Electropherograms of triplet-primed PCR of the *ATXN2* CAG repeat. (**B**) Electropherograms of multiplex PCR of four linked microsatellite markers flanking the *ATXN2* CAG repeat, performed on 10 ng parental genomic DNAs and whole genome amplified trophectoderm biopsy samples. (**C**) Inherited paternal and maternal marker haplotypes of embryos. The paternal high-risk haplotype associated with the expanded allele is highlighted in dark blue. The unaffected embryo is represented by an empty diamond symbol and affected embryos by filled diamond symbols. TE, trophectoderm; m, maternal allele; p, paternal allele.

In the second clinical IVF-PGT-M case, the affected husband carries one normal 22-repeat allele and one expanded 37-repeat allele, as does his affected mother, while his unaffected wife carries two normal 22-repeat alleles (Figure 3). During their first IVF-PGT cycle, TP-PCR of the single biopsied embryo showed one normal 22-repeat allele and one expanded 38-repeat allele, indicating inheritance of the paternal expanded allele. This embryo was diagnosed as affected, and no transfer was performed. In the second IVF-PGT cycle, four embryos were biopsied, and TP-PCR identified three affected embryos with CAG repeat genotypes of 22/45 (embryo #2), 22/45 (embryo #3), and 22/36 (embryo #4), and one unaffected embryo with homozygous 22 repeats (embryo #5). Microsatellite haplotyping corroborated these diagnoses, with affected embryos inheriting the paternal high-risk haplotype and the unaffected embryo inheriting the paternal low-risk haplotype. Transfer of the only unaffected embryo resulted in a successful pregnancy and live birth of a healthy infant.

### 2.2. Identification of Microsatellite Markers for Heptadecaplex PCR

In each IVF-PGT-M case, linkage analysis involved first searching for markers that were informative for the couple. Marker informativeness varies among couples, which means that markers that were informative in a previous couple may not be informative in other couples, and new markers may need to be identified and tested for informativeness in every prospective IVF-PGT-M case. This process is labour- and time-intensive. In order to simplify the process of identifying informative markers in future prospective couples wishing to undergo IVF-PGT-M for SCA2, we screened markers closely flanking the *ATXN2* CAG repeat to identify those exhibiting high polymorphic information content (PIC), observed heterozygosity (H_o_) and expected heterozygosity (H_e_) in two ethnically distinct population samples.

Through in silico mining, 463 microsatellite markers lying within 1.65 Mb upstream and downstream of the *ATXN2* CAG repeat were initially identified. Following additional filtering based on the previously described criteria [22,23] and subsequent manual curation, 287 markers remained.

Specifically, in silico filtering criteria required minimum repeat copy numbers based on nucleotide length, such that dinucleotide repeats contained 18 or more repeat units, trinucleotide repeats contained 12 or more repeat units, tetranucleotide repeats contained 10 or more repeat units, pentanucleotide repeats contained 8 or more repeat units, with all shortlisted markers demonstrating a minimum sequence match of 80%.

During preliminary screening of 46 candidate markers across 16 anonymized genomic DNA samples, 29 markers were excluded due to various reasons. Of these, 19 markers showed low PIC and heterozygosity values (Chr12:112897, Chr12:112877, Chr12:112849, Chr12:112630, Chr12:112510, Chr12:112387, Chr12:112263, Chr12:112247, Chr12:112200, Chr12:111942, Chr12:111761, Chr12:111381, Chr12:111141, Chr12:111100, Chr12:111061, Chr12:111058, Chr12:110811, Chr12:110693, and Chr12:110092), six markers displayed weak amplification (Chr12:112727, Chr12:112662, Chr12:111429, Chr12:111328, Chr12:111033, and Chr12:110593), three markers exhibited poor peak resolution which affected allele calling (Chr12:112471, Chr12:111394, and Chr12:110436), and one marker showed incompatibility in a highly multiplexed PCR panel (Chr12:112530).

The remaining 17 markers exhibited high polymorphism potential and compatibility for single-tube co-amplification. Eight of the 17 markers are located upstream of the *ATXN2* CAG repeat (Chr12:113048, Chr12:112791, Chr12:112765, Chr12:112694, Chr12:112667, Chr12:111939, Chr12:111935, and AFMA154TC5), and nine are located downstream (Chr12:111483, Chr12:111464, Chr12:111380, Chr12:111336, Chr12:110517, Chr12:110302, Chr12:110228, Chr12:110121, and D12S161). Two of the 17 markers are known (AFMA154TC5 and D12S161) while the remaining 15 are novel. This panel of 17 markers provides comprehensive coverage flanking the *ATXN2* CAG repeat, with AFMA154TC5 (~0.28 Mb upstream) and Chr12:111483 (~0.12 Mb downstream) representing the closest markers, while Chr12:113048 (~1.45 Mb upstream) and D12S161 (~1.63 Mb downstream) are the most distal markers relative to the repeat. Figure 1D shows a representative heptadecaplex PCR electropherogram result from one of the analyzed samples. Different fluorophore-labelled M13-tagged primers enable clear differentiation between markers based on peak colour and fragment size range.

### 2.3. Analysis of Marker Heterozygosity and Polymorphic Information Content

To evaluate marker heterozygosity and polymorphism, 190 DNA samples (94 Chinese and 96 Caucasian) were genotyped using the optimized heptadecaplex microsatellite marker panel. From the individual genotypes obtained, allele frequencies along with expected heterozygosity (H_e_), observed heterozygosity (H_o_), and PIC values were calculated for each marker. There were a total of 214 unique alleles observed across the 17 markers, where between 5 and 35 alleles were observed for each marker. The allele frequencies of the markers ranged from 0.005 to 0.91 (Figure S1 and Table S2). Each marker exhibited allelic variations specific to each population sample. For example, allele 175 of Chr12:111939 and allele 338 of Chr12:111935 were exclusive to the Chinese population sample, while allele 223 of Chr12:111939 and allele 310 of Chr12:111935 were only found in the Caucasian population sample.

**Figure 4:**
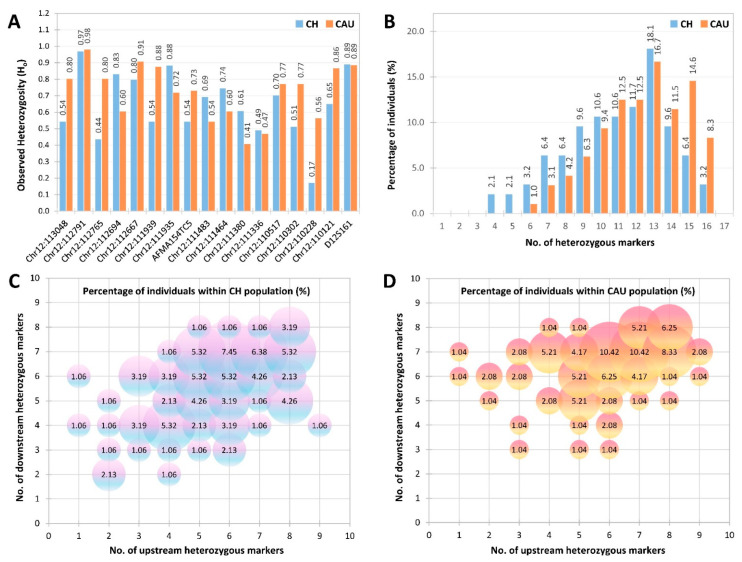
Heterozygosity analysis of 17 microsatellite markers flanking the *ATXN2* CAG repeat in Chinese (*n* = 94) and Caucasian (*n* = 96) population samples. (**A**) Observed heterozygosity of each marker, (**B**) percentage heterozygous for different numbers of markers, (**C**) percentage of Chinese individuals heterozygous for different numbers of upstream and downstream markers, and (**D**) percentage of Caucasians heterozygous for different numbers of upstream and downstream markers. CH, Chinese; CAU, Caucasian.

The H_e_, H_o_, and PIC values of the panel markers in the Chinese population samples range from 0.16–0.93, 0.17–0.97 and 0.15–0.92, respectively, while the corresponding value ranges in the Caucasian population samples are 0.41–0.93, 0.41–0.98 and 0.39–0.92, respectively (Figure 4A). In both populations, marker Chr12:112791 was found to be the most polymorphic with an H_o_ of 0.97 in the Chinese and H_o_ of 0.98 in the Caucasians. The panel of 17 microsatellite markers demonstrated overall high heterozygosity, with 100% of Chinese and Caucasian individuals heterozygous for at least six markers. Notably, a majority of individuals in both populations were heterozygous for 12 to 14 markers, with individuals heterozygous for 13 markers the most abundant group in both Chinese (17.0%) and Caucasian (16.7%) populations (Figure 3B).

Importantly, 100% of Chinese and Caucasian individuals were heterozygous for at least two markers on each side of the *ATXN2* CAG repeat (Figure 4C,D). This indicates that the heptadecaplex panel is highly polymorphic and informative in both populations, suggesting that enough markers for linkage-based PGT-M of SCA2 can be consistently identified from this single microsatellite marker panel without the need for additional couple-specific marker identification.

### 2.4. Validation of Heptadecaplex Panel on Whole Genome Amplified (WGA) Products

**Figure 5:**
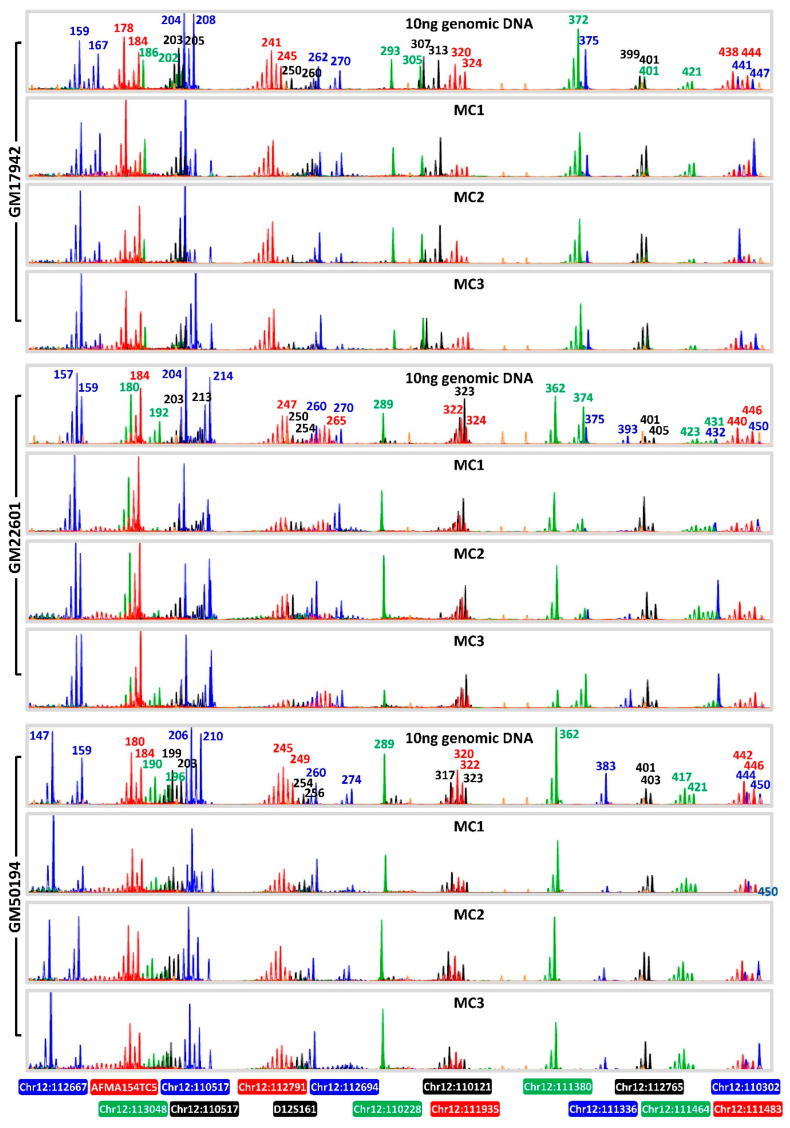
Successful single-tube amplification of SCA2 heptadecaplex microsatellite marker panel from five-cell samples, after whole genome amplification using multiple displacement amplification. ADO, allele dropout; MC1-MC3, triplicate five-cell samples that underwent whole genome amplification.

To validate the applicability of the heptadecaplex panel under conditions representative of PGT-M, the heptadecaplex multiplex PCR was also performed on whole genome amplified products of five-cell samples, which mimics the WGA product from embryonic biopsy samples. Biological triplicates from three different Coriell cell lines underwent WGA by multiple displacement amplification, and the heptadecaplex marker panel PCR was performed on an aliquot of the WGA product. The genotyping results obtained from the WGA products were compared against the corresponding genomic DNA results for each cell line. Electropherograms generated from the five-cell WGA products closely resembled those from 10 ng genomic DNA, demonstrating consistent allele peak patterns (Figure 5).

Accuracy of the microsatellite genotyping was evaluated by comparing amplification failure (AF) and allele dropout (ADO) rates between genomic DNA and WGA products. Genomic DNAs for all three cell lines had no AF nor ADO and were therefore used as references for evaluating the five-cell WGA products. The results from the five-cell WGA samples indicated comparable performance, achieving 100% amplification success rate, with only two ADOs observed out of 306 alleles assessed (Table S3).

## 3. Discussion

Currently, the primary method for detecting *ATXN2* CAG repeat expansion in SCA2 involves standard flanking PCR followed by capillary electrophoresis [9]. However, as with other trinucleotide repeat disorders, expanded *ATXN2* alleles cannot be reliably amplified using standard flanking PCR. This is because large alleles may fail to amplify due to preferential amplification of the shorter normal allele. This constraint is compounded by the limited DNA available in PGT-M, which is somewhat alleviated through whole genome amplification, although WGA itself may create biases of its own. Given that expansions in SCA2 may exceed 200 repeats, conventional PCR alone is unsuitable for reliable clinical application in PGT-M. When standard PCR is used, diagnosis relies on comparison of the couple’s normal alleles. This can be informative if the affected partner carries a normal allele of a different size from their unaffected partner’s normal alleles. However, when the couple’s normal alleles are identical, i.e., uninformative, it is not possible to confirm biparental inheritance of the normal allele in the embryo.

To overcome these limitations, TP-PCR was employed for the direct detection of the SCA2 repeat expansion. TP-PCR has been widely integrated into PGT-M workflows for other repeat expansion disorders and can likewise be applied in SCA2 PGT-M to reduce the risk of misdiagnosis. TP-PCR amplifies the *ATXN2* CAG repeat sufficiently from MDA whole genome amplified trophectoderm samples to discriminate between normal and expanded alleles, thereby overcoming the limitations of standard PCR.

In the first clinical SCA2 IVF-PGT-M case, TP-PCR was complemented with a customized panel of six microsatellite markers (four upstream and two downstream of the *ATXN2* CAG repeat) to establish the maternal high-risk haplotype in the embryos due to the absence of an index case. In the second case, a customized panel of four microsatellite markers (one upstream and three downstream) was used alongside TP-PCR to confirm inheritance of the paternal high-risk haplotype. In both cases, concordant TP-PCR and microsatellite marker results supported the transfer of an unaffected embryo, resulting in successful pregnancies and live births.

To simplify and rapidly identify informative markers for future SCA2 PGT-M cases, a panel of 17 highly polymorphic microsatellite markers closely flanking the *ATXN2* CAG repeat was selected to efficiently co-amplify in a single-tube PCR reaction. All 17 markers lie within 1.65 Mb of the *ATXN2* CAG repeat. The heptadecaplex marker panel precludes the need for couple-specific customized panels to identify informative markers, thereby reducing the time required for pre-clinical assay workup. The markers included in the heptadecaplex panel were selected for their high heterozygosity across two populations, ensuring a high probability of identifying at least one informative marker on each side of the *ATXN2* CAG repeat. As all markers are located within 1.65 Mb of the gene, the likelihood of meiotic recombination between the repeat and any individual marker is estimated to be <2%. This design aligns with the ESHRE PGT-M Working Group guidelines, which recommend that at least one fully informative microsatellite marker should flank each side of the locus of interest, in conjunction with detection of the pathogenic variant from low-template WGA samples [24]. By providing multiple heterozygous markers closely linked to the *ATXN2* pathogenic variant, this panel minimizes the risk of misdiagnosis due to ADO and complements TP-PCR to provide both direct and indirect evidence for accurate diagnosis.

Together, TP-PCR and the heptadecaplex marker panel serve as complementary tools for rapid and reliable IVF-PGT-M of SCA2 in at-risk couples.

Despite these strengths, however, there are several limitations of this study. Firstly, although population-level screening demonstrated high PIC and heterozygosity of the panel, the number and diversity of populations evaluated were limited. Additional population screening across more ethnic groups will be required to confirm the universal applicability of the panel across diverse genetic backgrounds.

Secondly, only two SCA2 PGT-M cases were described in this study. Between these two cases, three microsatellite markers were used in common; however, there were differences observed in marker informativeness between the two couples. In the first case, marker Chr12:111935 was only partially informative, which required the use of additional telomeric markers, while centromeric markers were sufficient.

In contrast, in the second case, Chr12:111935 was fully informative, whereas centromeric markers Chr12:111380 and Chr12:110517 were only partially informative, and other markers also showed partial informativeness.

Although insufficient for population-level generalizability, these observations illustrate variability in marker informativeness that may occur in different couples undergoing PGT-M. Evaluation across a larger number of cases will be required to determine the broader performance characteristics of the panel.

In the unlikely event that all 17 markers in this panel are uninformative for a particular couple, the other markers identified in this study (Supplementary Table S1) provide a useful resource for additional markers to be evaluated.

Thirdly, although only markers within 1.65 Mb of the *ATXN2* gene were selected to minimize recombination risk, meiotic recombination between the closest flanking marker and the CAG repeat cannot be completely excluded. This is an inherent limitation of linkage-based approaches.

Fourthly, TP-PCR is not completely immune to preferential amplification and stutter artefacts, which may affect precise sizing, especially of very large expansions, despite the TP-PCR assay design of a taller terminal peak to improve allele sizing accuracy.

Finally, validation of the marker panel was performed using WGA products derived from multicell cell lines rather than actual trophectoderm embryo biopsy samples, which may not fully reflect the performance of the heptadecaplex panel in real clinical settings.

## 4. Materials and Methods

### 4.1. Biological Samples

Genomic DNA from two couples undergoing IVF-PGT-M was analyzed to confirm the presence of an *ATXN2* CAG repeat expansion in the affected partner. Embryos from both couples were generated through intracytoplasmic sperm injection of the retrieved oocytes.

For the first couple, a single IVF-PGT-M cycle was performed in which twelve oocytes were retrieved and fertilized, resulting in seven blastocysts biopsied on day 5 (*n* = 6) and day 6 (*n* = 1), with two to seven trophectoderm cells retrieved from each embryo. The second couple underwent two IVF-PGT-M cycles. In the first cycle, four of seven retrieved oocytes fertilized and a single blastocyst was biopsied on day 6, with five to seven cells collected. In the subsequent cycle, five of eight retrieved oocytes fertilized, resulting in four day-5 blastocysts biopsied, with three to five cells collected from each embryo.

Each biopsy sample was incubated in 1.5 µL of lysis buffer (0.6 mol/L KOH), heated at 30 °C for 10 min, rapidly cooled to 4 °C, and neutralized with 1.5 µL of 0.6 mol/L Tricine. WGA was performed using GenomiPhi V2 DNA Amplification Kit (Cytiva-Danaher, Buckinghamshire, UK) according to the manufacturer’s instructions, except that the incubation time was 4 h. A 2 µL aliquot of WGA product was used as template in each *ATXN2* TP-PCR and customized multiplex microsatellite PCR assay.

Genomic DNA of 16 cell lines purchased from Coriell Cell Repository (CCR) (Camden, NJ, USA) was used for initial screening of potential microsatellite markers and optimization of a comprehensive multiplex PCR panel. To evaluate the PIC, H_e_, and H_o_ of each marker, a total of 190 DNA samples were analyzed. These included 94 anonymized cord blood samples from unrelated Chinese newborns at the National University Hospital, Singapore, and 96 Caucasian DNA samples from the Human Variation Panel (HD100CAU, CCR).

For testing of the heptadecaplex microsatellite PCR panel on WGA products, five-cell samples were manually isolated from cell lines GM17942, GM22601 and GM50194, and underwent the same WGA process as the embryonic biopsy samples. A 2 µL aliquot of WGA product was used as template in the heptadecaplex microsatellite PCR assay.

### 4.2. PGT-M for SCA2 Using ATXN2 (CAG)_n_ TP-PCR and Linked Microsatellite Analysis

With written informed consent, both couples were offered and chose to undergo PGT-M for SCA2 as the affected spouse in each case had a confirmed family history of the condition. Testing of the *ATXN2* (CAG)_n_ TP-PCR assay for the first couple was initially performed using 10 ng of parental genomic DNA and subsequently validated on the WGA product generated from single cells from cell line GM04866 (CCR). For the second couple, validation was similarly performed using the WGA product generated from 100 pg of the affected spouse’s genomic DNA.

The TP-PCR assay consisted of 0.2 μmol/L HEX-SCA2-F, 0.02 μmol/L SCA2-5′TP-R, and 0.2 μmol/L Tail primers (Table 1) and was performed in a 50 µL reaction containing 2–2.5 U of HotStarTaq DNA polymerase (Qiagen, Hilden, Germany), 0.5× Q-solution (Qiagen), 1× PCR buffer containing 1.5 mmol/L MgCl_2_ (Qiagen), deoxynucleotide triphosphate (dNTP) mix consisting of 0.2 mmol/L each of dATP, dTTP, dCTP, and dGTP (Roche Applied Science, Penzberg, Germany), and 2 µL of WGA product of trophectoderm biopsy sample. Primer HEX-SCA2-F was fluorescently labelled at the 5′ end with hexachlorofluorescein (HEX). Thermal cycling involved a 15 min enzyme activation at 95 °C, followed by 35 cycles of denaturation at 98 °C for 45 s, annealing at 60 °C for 1 min, extension at 72 °C for 2 min, and a final extension at 72 °C for 5 min, on the SimpliAmp thermal cycler (Applied Biosystems-Thermo Fisher Scientific, Foster City, CA, USA).

A 1 µL aliquot of the fluorescently labelled PCR product was combined with 9 μL of Hi-Di™ formamide (Applied Biosystems) and 0.2–0.5 μL of size standard GeneScan™ 500 ROX™ dye (Applied Biosystems), denatured at 95 °C for 5 min, rapidly cooled to 4 °C, and resolved across a 36 cm capillary filled with POP-7™ polymer in a 3130*xl* Genetic Analyzer or a 28 cm capillary filled with POP-1™ polymer in a SeqStudio Genetic Analyzer (Applied Biosystems). Samples were electrokinetically injected at 1 kV for 15 s and electrophoresed for 40 min at 60 °C in the 3130*xl* Genetic Analyzer, or injected at 1.2 kV for 7 s and electrophoresed for 20 min in the SeqStudio Genetic Analyzer. Data analysis was performed using GeneMapper software version 6.0 (Applied Biosystems).

*ATXN2* TP-PCR was supplemented with haplotype analysis of linked microsatellite markers to establish whether each embryo had inherited the high- or low-risk parental haplotype. Markers within 1.65 Mb upstream and downstream of the *ATXN2* gene were retrieved from ‘Microsatellite’ and ‘STS Markers’ tracks in the UCSC Genome Browser (https://genome.ucsc.edu/). A total of six informative markers were identified for the first couple and selected for haplotype analysis, with four upstream (Chr12:112791, Chr12:112667, Chr12:112387, and Chr12:111935) and two downstream (Chr12:111380 and Chr12:110517). As for the second couple, four informative markers were selected, consisting of one upstream marker (Chr12:111935) and three downstream markers (Chr12:111483, Chr12:111380, and Chr12:110517).

Multiplex-PCR of the respective customized microsatellite marker panels was performed in a 50 µL reaction containing 2.5 U of HotStarTaq DNA polymerase, 1× PCR buffer containing 1.5 mmol/L MgCl_2_, dNTP mix consisting of 0.2 mmol/L each of dATP, dTTP, dCTP, and dGTP, 0.2–0.4 µmol/L of primers, and 2 µL of WGA product of trophectoderm biopsy sample. One primer from each primer pair was tailed at its 5′ end with either the M13-1 or M13-2 bacteriophage M13 sequence. Thermal cycling involved a 15 min enzyme activation at 95 °C, followed by 30 cycles of denaturation at 98 °C for 45 s, annealing at 55 °C or 60 °C for 1 min, extension at 72 °C for 1 min, and a final extension at 72 °C for 5 min, on the SimpliAmp thermal cycler.

A 2 µL aliquot of each PCR product was subjected to an extension labelling reaction in a 20 µL volume using 0.1 µmol/L each of the 6-FAM-labelled M13-1 primer and HEX-labelled M13-2 primer. Thermal cycling was identical to the multiplex PCR, except that five cycles were performed.

A 1 µL aliquot of the fluorescently labelled PCR product was mixed with 9 μL of Hi-Di™ formamide and 0.2–0.3 μL of GeneScan™ 500 ROX™ dye size standard. Samples were prepared for capillary electrophoresis as described above. Samples were electrokinetically injected at 1.2 kV for 23 s and electrophoresed for 20 min at 60 °C in the 3130*xl* Genetic Analyzer, or injected at 1.2 kV for 7 s and electrophoresed for 20 min in the SeqStudio Genetic Analyzer. GeneScan analysis was performed using GeneMapper software version 6.0. For the first couple, there was no available index case, so the maternal high-risk marker haplotype linked to the expanded *ATXN2* allele was established during the actual clinical cycle. In contrast, for the second couple, the husband’s mother was clinically affected, and her genotype was used to determine the paternal high-risk marker haplotype.

### 4.3. Development of Heptadecaplex Microsatellite Marker Panel

DNA sequences spanning up to 1.65 Mb upstream and downstream of the *ATXN2* gene were retrieved from the National Center for Biotechnology Information (NCBI, http://www.ncbi.nlm.nih.gov/) using accession number NC_000012.12 (GRCh38.p13 Primary Assembly). Microsatellites within these regions were identified using the Tandem Repeat Finder (TRF) DNA analysis program. The identification, selection, and primer design for microsatellite markers have been previously described [22,23]. To assess preliminary PIC and heterozygosity values, PCR primer pairs were designed for each candidate microsatellite and tested on 16 DNA samples isolated from Coriell Cell Repositories cell lines. One primer in each pair was 5′ end-tailed with M13-1, M13-2, M13-3, or M13-4 sequences derived from bacteriophage M13.

Based on preliminary genotyping results from these 16 different DNA samples, markers that exhibited low PIC and heterozygosity values, poor amplification, or non-distinct and ambiguous peak patterns were excluded. A total of 17 microsatellite markers were eventually selected for further co-amplification and optimization in a single-tube multiplex reaction (Table 2).

### 4.4. Singleplex and Heptadecaplex Microsatellite PCR Amplification

The initial testing of each individual microsatellite marker was performed in a 50 µL reaction volume consisting of 10 ng genomic DNA, 2.5 U HotStarTaq DNA polymerase, 1× PCR buffer containing 1.5 mmol/L MgCl_2_, dNTP mix consisting of 0.2 mmol/L each of dATP, dTTP, dCTP, and dGTP, and 0.2 µmol/L of both forward and reverse primers. Thermal cycling involved an initial 15 min enzyme activation at 95 °C, 30 cycles of denaturation at 98 °C for 45 s, annealing at 60 °C for 1 min, and extension at 72 °C for 1 min, and a final extension at 72 °C for 5 min.

The heptadecaplex microsatellite PCR was performed in a 25 µL reaction consisting of 1× Platinum^TM^ Multiplex PCR Master Mix (Applied Biosystems-Thermo Fisher Scientific, Foster City, CA, USA), 0.1–0.2 µmol/L of primers (Table 2), and either 10 ng of genomic DNA or 2 µL of single cell WGA product as template. Thermal cycling involved a 2 min enzyme activation at 95 °C, followed by 30 cycles of denaturation at 95 °C for 30 s, annealing at 60 °C for 1 min 30 s, extension at 72 °C for 1 min with an increment of 6 s after every cycle, and a final extension at 60 °C for 30 min, on the GeneAmp 9700 thermal cycler (Applied Biosystems-Thermo Fisher Scientific, Foster City, CA, USA).

To visualize the amplification products, 2 µL of the PCR product was subjected to extension labelling in a 20 µL reaction using 0.2 µmol/L each of the 6-FAM-labelled M13-1 primer, VIC-labelled M13-2 primer, NED-labelled M13-3 primer, and PET-labelled M13-4 primer. Thermal cycling was identical to the heptadecaplex PCR, except that 10 cycles were performed, with no 6 s increments after every cycle.

### 4.5. Heptadecaplex Microsatellite Genotyping

An aliquot of 2.5 μL fluorescently labelled PCR product was mixed with 9 μL of Hi-Di™ Formamide and 0.3 μL of GeneScan™ 500 LIZ™ dye size standard (Applied Biosystems). The mixed solution was denatured at 95 °C for 5 min and cooled to 4 °C before it was resolved in a SeqStudio Genetic Analyzer using a 28 cm capillary filled with POP-1™ polymer. Samples were electrokinetically injected at 1.2 kV for 7 s and electrophoresed for 20 min at 60 °C. Post-electrophoresis fragment analysis was performed using GeneMapper software version 6.1 (Applied Biosystems).

### 4.6. Data Analysis

Allele frequency, H_e_ [25], H_o_ [26], and PIC [27], of the 17 microsatellite markers in the comprehensive panel were calculated using Microsoft Excel.

## Figures and Tables

**Table 1:** *ATXN2* (CAG)_n_ TP-PCR primers and expected product sizes.

Primer	Primer Sequence (5′-3′)	GenBank ID: Nucleotides	Concentration (µmol/L)	Expected TP-PCR Product Size
HEX-SCA2-F	HEX-GTGCGAGCCGGTGTATGG	NG_11572: 5615–5632	0.20	240 bp + (CAG)_n_
SCA2-5′TP-R	GTTTCGGCGTTACGAGTGGA**CGG**(CTG)_5_	-	0.02	
Tail5	GTTTCGGCGTTACGAGTGGA	-	0.20	

Bolded and underlined nucleotides are complementary to the immediate downstream flanking sequence of the repeat locus.

**Table 2:** Heptadecaplex microsatellite marker PCR details.

Microsatellite Marker	Repeat Motif	Primer Sequence (5′-3′) ^a^		Concentration (µmol/L)	Amplicon Size Range (bp) ^d^	H_e_ ^d^		H_o_ ^d^		PIC ^d^	
						CH	CAU	CH	CAU	CH	CAU
**Chr12:113048**	(GT)n	F	^M13−2^ CCAGAGAAAGAACGGGGTTTAGAG	0.15	180–200	0.59	0.78	0.54	0.80	0.52	0.76
		R	^b^ CAAATGAACCAGCTCTGCATAAGC	0.15							
**Chr12:112791**	(TC)n	F	^M13−4^ CACATGGATGAAGGTTTTAGCTCTG	0.15	207–259	0.93	0.93	0.97	0.98	0.92	0.92
		R	^b^ CTTGCTCACACAGGGCTTTG	0.15							
**Chr12:112765**	(AC)n	F	^M13−3^ ATAGTGGGGAACTGTGGGGTC	0.15	395–409	0.44	0.75	0.44	0.80	0.41	0.71
		R	^b^ CTGGTGTGAGAGAGGAATGCC	0.15							
**Chr12:112694**	(TG)n/(TC)n	F	^M13−1^ CATGGAAGAAGAGGCCATTTCAAG	0.2	250–276	0.78	0.62	0.83	0.60	0.75	0.56
		R	^b^ CACCAAGCTGAGATTGTCATGC	0.2							
**Chr12:112667**	(TC)n	F	^M13−1^ GACTTCAGTGATCCTCCAAGGTG	0.15	147–177	0.78	0.86	0.80	0.91	0.76	0.85
		R	^c^ TCACCATTCATGGGCAGAGATG	0.15							
**Chr12:111939**	(AC)n	F	^M13−3^ TTAGTCTGCTGTTGTAGTAGCTCC	0.15	175–223	0.51	0.85	0.54	0.88	0.49	0.84
		R	^c^ TGATCATATTACTGCACTCCAGCG	0.15							
**Chr12:111935**	(AC)n	F	^b^ GAGGTCACAGTAAGCTGAGATCG	0.15	306–342	0.88	0.70	0.88	0.72	0.85	0.68
		R	^M13−4^ TCTGAATATACACTGAAACCTGGGC	0.15							
AFMA154TC5	(TG)n	F	^M13−4^ GACGAAATCATCAGAAGGCTTCATAGG	0.15	166–190	0.53	0.73	0.54	0.73	0.48	0.69
		R	^b^ GATGTATACTTCCTGATCGTTTTCTGGG	0.15							
**Chr12:111483**	(TG)n	F	^M13−4^ GTGCCCAATAATTGCCTTTCTGAC	0.2	414–450	0.68	0.58	0.69	0.54	0.65	0.55
		R	^c^ TCAAACATGGAGAAAATTGGCTGG	0.2							
**Chr12:111464**	(AC)n	F	^M13−2^ GGAAATACCCAATTTAAGAAGGCTGG	0.2	411–427	0.67	0.56	0.74	0.60	0.63	0.54
		R	^b^ CTTTAGCCATGCACAGGACTG	0.2							
**Chr12:111380**	(GT)n	F	^M13−2^ GCACCACACATCTATTCTAATGGG	0.1	360–376	0.61	0.41	0.61	0.41	0.57	0.39
		R	^b^ GCTGTCAGCAAACTCAGATCCC	0.1							
**Chr12:111336**	(AC)n	F	^b^ AAACTGAAGTGTGGGCTGGG	0.2	361–393	0.43	0.45	0.49	0.47	0.40	0.42
		R	^M13−1^ CCATTGGCCTCTCAGACACTTC	0.2							
**Chr12:110517**	(TG)n	F	^M13−1^ CCCTACATACCATTTCATAATCTACCC	0.15	194–216	0.71	0.73	0.70	0.77	0.68	0.69
		R	^b^ GGTATGTATCAGAGAGGTTTAATGAAGC	0.15							
**Chr12:110302**	(AAT)n	F	^b^ GGGCTTAACTAAAATAATAATAGGCCAGGC	0.2	441–453	0.49	0.66	0.51	0.77	0.43	0.60
		R	^M13−1^ TCTTCCCCCACAGTATGGTAATTGC	0.2							
**Chr12:110228**	(AAAT)n	F	^b^ GAATCAAATAATAAAATGAGGCTGGGCGTG	0.1	285–309	0.16	0.50	0.17	0.56	0.15	0.47
		R	^M13−2^ CAGGTGGTAAGCCAGTTAATTTGTTTC	0.1							
**Chr12:110121**	(TG)n	F	^M13−3^ AAGGAAGATTGGCTACCACCC	0.15	309–327	0.72	0.81	0.65	0.86	0.68	0.79
		R	GTTGCTGACAATTCCCTCTTAGAC	0.15							
D12S161	(GT)n	F	^M13−3^ GAGATTGCCCTGCTTCCCATC	0.15	232–272	0.89	0.90	0.88	0.89	0.88	0.89
		R	^b^ CCGCTACCTACACTGGTCAG	0.15							

Markers highlighted in bold are novel. ^a^ Primer sequences were based on genome assembly build GRCh38/hg38. ^b^ 5′ GTTT tailed; ^c^ 5′ GTT tailed;. ^M13−1^, 5′ GGTTTTCCCAGTCACGAC tailed; ^M13−2^, 5′ GTAAAACGACGGCCAGTG tailed; ^M13−3^, 5′ CATGGTCATAGCTGTTTCCTG tailed; ^M13−4^, 5′ CCGCTAATTCAACCCATTGC tailed. ^d^ Amplicon size, H_e_ (expected heterozygosity), H_o_ (observed heterozygosity), and PIC (polymorphism information content) were determined from 94 CH (Chinese) and 96 CAU (Caucasian) DNA samples.

## Data Availability

The original contributions presented in this study are included in the article/Supplementary Material. Further inquiries can be directed to the corresponding author.

## Supplementary Materials

The following supporting information can be downloaded at: https://www.mdpi.com/article/10.3390/ijms27031546/s1.
