# Supplementary material for: Preimplantation Genetic Testing of Spinocerebellar Ataxia Type 2—Robust Tools for Direct and Indirect Detection of the ATXN2 CAG Repeat Expansion
Source: Int J Mol Sci. 2026 Feb 4;27(3):1546. doi: 10.3390/ijms27031546 (PMC12898808; doi:10.3390/ijms27031546)
Supplement: Supplementary file 1 [file ijms-27-01546-s001.zip › ijms-4079287-supplementary.pdf]

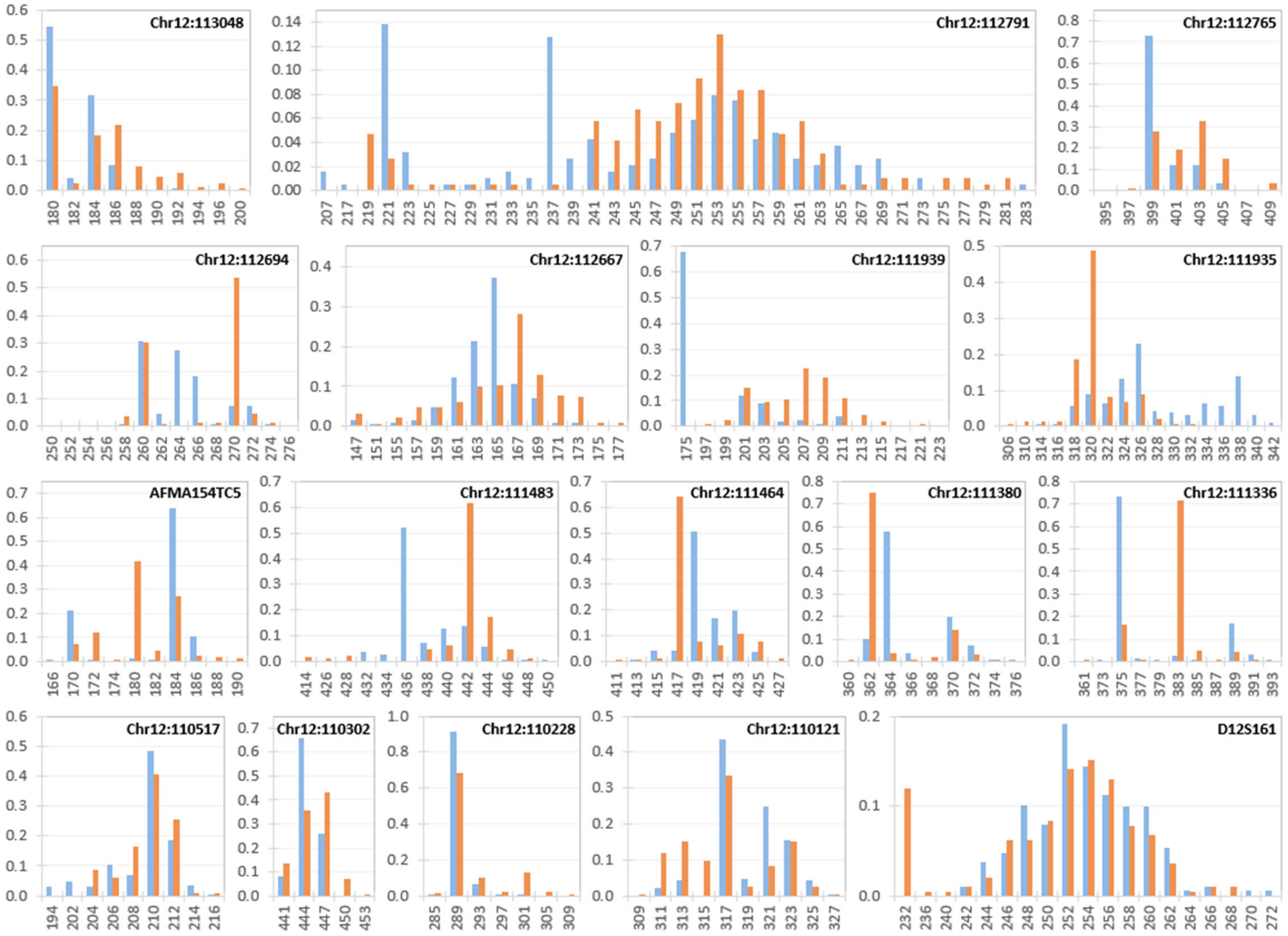

Figure S1. Allele frequencies of each of the 17 microsatellite markers in the Chinese and Caucasian populations. CH, Chinese; CAU, Caucasian.

Table S1. *In silico* mining of microsatellite markers located ~1.5 Mb upstream and ~1.65 Mb downstream of *ATXN2* $(CAG)_n$ on chromosome 12q24.12, identified using Tandem Repeat Finder (TRF).

Microsatellite marker	Marker location	Repeat Motif	No. of repeats	Percentage of matches	Score
Chr12:113096	113096502-113096443	TTTTC	12.8	87	67
Chr12:113088	113088865-113088840	AAC	8.7	100	52
Chr12:113080	113080627-113080590	TTCCA	7.6	93	67
Chr12:113079	113079737-113079695	AC	21.5	100	86
Chr12:113062	113062070-113062033	GT	19	100	76
Chr12:113053	113053388-113053359	CATC	7.5	92	51
**Chr12:113048**	**113049031-113048990**	**GT**	**21**	**95**	**75**
Chr12:113047	113047542-113047476	TTTC	16.2	98	116
Chr12:113047	113047458-113047415	TCCT	11	100	88
Chr12:113045	113045986-113045917	TCC	23.3	85	68
Chr12:113045	113045889-113045855	AC	17.5	93	61
Chr12:113044	113044899-113044831	TCCCT	14.2	89	106
Chr12:113044	113044287-113044255	TC	16.5	93	57
Chr12:113039	113039050-113038780	TCTT	67.5	88	318
Chr12:113036	113036369-113036343	TTTG	6.8	100	54
Chr12:113012	113012639-113012608	TTTTC	6.8	96	50
Chr12:113005	113005736-113005703	TTTA	8.8	93	61
Chr12:113001	113001545-113001352	AT	97	80	82
Chr12:112972	112972913-112972880	AC	17	100	68
Chr12:112954	112954307-112954282	AAC	8.7	100	52
Chr12:112942	112942832-112942766	AAT	22.3	83	66
Chr12:112935	112935125-112935096	AAAT	7.2	96	51
Chr12:112934	112934605-112934485	TTCT	29	84	131
Chr12:112912	112912366-112912339	TTTG	7	100	56
Chr12:112897	112897175-112897146	GT	15	100	60
AFM291XE9	112877459-112877404	GT	28	92	85
AFMA219ZG5	112856513-112856485	AC	14.5	100	58
Chr12:112849	112849456-112849409	AC	24	86	69
Chr12:112803	112803512-112803487	TAA	8.7	100	52
**Chr12:112791**	**112791905-112791868**	**TC**	**19**	**100**	**76**
Chr12:112781	112781718-112781674	ATGA	11.2	82	54
Chr12:112781	112781227-112781163	TTTTG	12.8	85	71
**Chr12:112765**	**112765285-112765247**	**AC**	**19.5**	**100**	**78**
Chr12:112759	112759243-112759210	AC	17	87	50
Chr12:112755	112755339-112755299	TG	20.5	100	82
Chr12:112730	112730033-112730003	TG	15.5	93	53
Chr12:112727.1	112727491-112727425	TG	33.5	87	98
Chr12:112727.2	112727062-112727024	TTTA	9.5	91	60
Chr12:112725	112725810-112725767	AAC	14.7	100	88
Chr12:112713	112713102-112712880	AAG	73.7	88	322
Chr12:112712	112712694-112712656	AC	19.5	89	60
Chr12:112694	112694684-112694645	TG	20	94	71

**Chr12:112694**	**112694618-112694552**	**TC**	**33.5**	**82**	**82**
Chr12:112678	112678389-112678233	TTTC	39	92	181
Chr12:112677	112677486-112677340	GGAA	36.8	91	154
**Chr12:112667**	**112667721-112667663**	**TC**	**29.5**	**100**	**118**
Chr12:112663	112663123-112663091	TC	16.5	93	57
Chr12:112663	112663088-112663060	CA	14.5	100	58
Chr12:112662	112662400-112662353	GGAT	12	86	71
Chr12:112647	112647626-112647591	AC	18	94	63
Chr12:112634	112634396-112634350	TTTG	11.8	84	60
Chr12:112630	112630189-112630120	TGGA	17.8	85	79
Chr12:112619	112619299-112619247	AC	26.5	100	106
Chr12:112605	112605404-112605374	TTTG	8	92	55
Chr12:112572	112572656-112572595	AG	32	85	74
Chr12:112572	112572561-112572429	AGAA	34	89	211
Chr12:112565	112565965-112565921	AAAAC	9	90	72
Chr12:112549	112549509-112549467	AC	21.5	100	86
Chr12:112543	112543399-112543365	AG	17.5	100	70
Chr12:112543	112543366-112543253	GAAG	28	83	61
Chr12:112543	112543281-112543221	GAAA	16	83	65
Chr12:112537	112537959-112537818	GAAG	35.5	84	191
Chr12:112534	112534036-112534002	AAAT	9	93	63
Chr12:112530	112530505-112530466	ATA	13.3	100	80
Chr12:112525	112525173-112525149	AC	12.5	100	50
Chr12:112517	112517168-112517121	TG	24	100	96
Chr12:112517	112517078-112517038	GT	20.5	100	82
Chr12:112510	112510392-112510353	TG	20	94	71
Chr12:112506	112506993-112506947	CAT	15.7	100	94
Chr12:112471	112471491-112471455	TC	18.5	100	74
Chr12:112459	112459942-112459909	GT	17	100	68
Chr12:112453	112453519-112453489	AAAT	7.8	100	62
Chr12:112442	112442889-112442831	TA	30	93	102
Chr12:112425	112425009-112424957	AC	26.5	100	106
Chr12:112424	112424919-112424868	CA	26	96	95
Chr12:112413	112413613-112413585	AAAAC	5.8	100	58
Chr12:112412	112412960-112412924	TATT	9.2	100	74
Chr12:112399	112399321-112399255	TTA	22.7	84	84
Chr12:112391	112391391-112391300	AT	46	82	76
Chr12:112387	112387157-112387129	TG	14.5	100	58
Chr12:112383	112383820-112383783	TTA	12.7	88	58
Chr12:112382	112382360-112382328	GGC	11	100	66
Chr12:112382	112382118-112382088	CGG	10.3	92	53
Chr12:112367	112367349-112367307	TTTC	10.8	100	86
Chr12:112335	112335180-112335144	TG	18.5	94	65
Chr12:112334	112334251-112334208	TTTA	11	100	88
Chr12:112328	112328155-112328111	AAAT	11.2	100	90
Chr12:112324	112324122-112323948	AAAG	43.5	95	143

Chr12:112311	112311470-112311438	TTTA	8.2	100	66
Chr12:112299	112299658-112299620	TTTG	10	94	71
Chr12:112274	112274733-112274706	TTTTG	5.6	100	56
Chr12:112263	112263757-112263725	TG	16.5	93	57
Chr12:112247	112248006-112247978	TG	14.5	100	58
Chr12:112246	112246176-112246142	ATTTT	7	100	70
Chr12:112240	112240466-112240435	AAAAG	6.8	96	50
Chr12:112229	112229363-112229328	GTTT	9	100	72
Chr12:112226	112226245-112226193	TG	26.5	88	79
Chr12:112217	112217357-112217301	TG	28.5	92	96
Chr12:112213	112213731-112213692	TA	20.5	89	64
Chr12:112212	112212813-112212789	AATA	6.2	100	50
Chr12:112207	112207157-112207117	ATAC	10.2	100	82
Chr12:112203	112203895-112203859	TATTT	7.6	85	51
Chr12:112200	112200912-112200850	ACAC	15.8	93	72
Chr12:112124	112124606-112124557	ATAAT	10	100	100
Chr12:112114	112114423-112114379	ATTT	11	90	72
Chr12:112086	112086103-112086074	GT	15	100	60
Chr12:112073	112073286-112073262	TG	12.5	100	50
Chr12:112057	112057241-112057190	TATTT	10.2	89	77
Chr12:112044	112044704-112044669	TGTTT	7.4	93	65
Chr12:112037	112037321-112037276	AT	23	100	92
Chr12:112034	112034660-112034629	TTG	10.7	100	64
Chr12:111982	111982657-111982633	TGAT	6.2	100	50
Chr12:111976	111976508-111976479	TGTTT	6	92	51
Chr12:111950	111950152-111950117	TTGTT	7.4	93	65
Chr12:111942	111942814-111942763	CA	26	84	68
Chr12:111940	111940584-111940499	AT	43	90	55
**Chr12:111939**	**111939935-111939881**	**AC**	**27.5**	**100**	**110**
**Chr12:111935**	**111935661-111935614**	**AC**	**24**	**100**	**96**
Chr12:111929	111929689-111929464	TTTC	54.8	88	141
Chr12:111919	111919220-111919190	TGTTT	6.2	100	62
Chr12:111918	111918815-111918781	GTTT	9	93	63
Chr12:111908	111908606-111908565	TTCC	10.5	100	84
Chr12:111894	111894792-111894767	CA	13	100	52
**AFMA154TC5**	**111887855-111887815**	**TG**	**20.5**	**100**	**82**
Chr12:111878	111878438-111878399	AAAT	10.2	94	73
Chr12:111859	111859666-111859628	AAAAG	7.8	91	51
Chr12:111765	111765037-111764983	AC	27.5	100	110
Chr12:111761	111761334-111761292	AC	21.5	95	77
Chr12:111752	111752310-111752275	AAC	12.3	88	56
Chr12:111737	111737869-111737842	AC	14	100	56
Chr12:111735	111735855-111735809	AAAT	11.8	82	60
Chr12:111722	111722408-111722370	AATA	10	94	71
Chr12:111720	111720801-111720754	AAAAT	9.8	80	55
Chr12:111704	111704691-111704665	AAAG	6.8	100	54

Chr12:111651	111651257-111651226	TAT	10.7	100	64
Chr12:111631	111631574-111631543	AAT	10.7	100	64
Chr12:111620	111620164-111620139	AATT	6.5	100	52
Chr12:111612	111612404-111612372	TTTTC	6.8	96	59
Chr12:111600	111600962-111600936	TTTA	6.8	100	54
ATXN2 (CAG)n	111599019-111598950	CAG	23.3	97	131
AFMB329WA5	111596246-111596206	TG	20.5	100	82
Chr12:111588	111588223-111588189	TTTA	8.8	100	70
Chr12:111577	111577282-111577257	AAAT	6.5	100	52
Chr12:111576	111576137-111576086	ATGTT	10.4	100	104
Chr12:111569	111569020-111568987	AAAAC	7	93	61
Chr12:111496	111496176-111496152	TTTG	6.2	100	50
Chr12:111489	111489799-111489762	TTTAT	7.6	82	51
Chr12:111484	111484449-111484411	AAAC	10	88	62
**Chr12:111483**	**111483239-111483189**	**TG**	**25.5**	**95**	**93**
**Chr12:111464.1**	**111464370-111464325**	**AC**	**23**	**95**	**83**
Chr12:111464.2	111464283-111464248	AC	18	100	72
Chr12:111463	111463002-111462978	TG	12.5	100	50
Chr12:111447	111447572-111447548	CCCCA	5	100	50
Chr12:111433	111433792-111433763	AAAAC	5.8	96	51
Chr12:111429	111429070-111429016	CCT	18.3	96	101
Chr12:111417	111417491-111417456	AAAT	8.8	93	63
Chr12:111415	111415959-111415923	TAAA	9.2	93	65
Chr12:111399	111399986-111399907	TCCT	19.5	92	133
Chr12:111395	111395319-111395281	AAT	13.3	94	71
Chr12:111394.1	111394475-111394444	TC	16	100	64
Chr12:111394.2	111394440-111394412	AT	14.5	100	58
Chr12:111386	111386525-111386496	TTTA	7.5	100	60
Chr12:111381	111381048-111381007	GT	21	90	66
**Chr12:111380**	**111380947-111380916**	**GT**	**16**	**100**	**64**
Chr12:111379	111379163-111379120	AC	22	100	88
Chr12:111355	111355965-111355933	AAAAC	6.6	92	57
**Chr12:111336**	**111336459-111336421**	**AC**	**20**	**94**	**71**
Chr12:111330	111330751-111330681	TA	35.5	97	133
Chr12:111329	111329005-111328942	GA	32	87	83
Chr12:111328	111328620-111328581	CA	20	100	80
Chr12:111318	111318087-111318050	AAC	12.7	88	58
Chr12:111310	111310030-111310006	CAGA	6.2	100	50
Chr12:111295	111295780-111295752	AATT	7.2	100	58
Chr12:111276	111276672-111276627	CAAAA	10	95	64
Chr12:111276	111276670-111276628	AAAC	9.8	97	50
Chr12:111265	111265358-111265292	AAAAT	13.2	96	125
Chr12:111263	111263659-111263626	TTTTG	6.8	100	68
Chr12:111259	111259087-111259032	CA	28	96	103
Chr12:111191	111191348-111191303	AAAT	11.8	95	85
Chr12:111179	111179620-111179594	AAC	9	100	54

Chr12:111167	111167705-111167670	AAAC	9	100	72
Chr12:111145	111145880-111145833	ACAAA	10.2	86	59
Chr12:111141	111141256-111141228	TG	14.5	100	58
Chr12:111137	111137396-111137350	AAC	15.3	82	58
Chr12:111134	111134649-111134621	AC	14.5	100	58
Chr12:111134	111134621-111134589	AG	16.5	93	57
Chr12:111100	111100232-111100182	AC	25.5	84	68
Chr12:111070	111070456-111070327	GAAG	32.2	89	172
Chr12:111069	111069571-111069521	AC	25.5	95	66
Chr12:111061	111061216-111061179	TG	19	100	76
Chr12:111058	111058595-111058536	CTCT	15	89	75
Chr12:111034	111034756-111034713	CCT	14.7	90	72
Chr12:111033	111034003-111033971	CGC	11.3	93	59
Chr12:111032	111032811-111032777	TTTG	8.8	100	70
Chr12:111019	111019111-111019077	AAAC	9	93	63
Chr12:111018	111018764-111018729	AAAC	9	88	56
Chr12:111018	111018291-111018180	CCCT	28	88	73
Chr12:111011	111011725-111011678	TCCT	12	95	87
Chr12:111000	111000995-111000967	CAA	9.7	100	58
Chr12:110969	110969384-110969346	AAAAG	7.8	97	69
Chr12:110968	110968478-110968448	TAAA	7.8	100	62
Chr12:110963	110963590-110963562	AAAAT	5.8	100	58
Chr12:110927	110927694-110927654	TG	20.5	89	55
Chr12:110917	110917521-110917491	TGTT	7.8	100	62
Chr12:110916	110916864-110916840	AAAT	6.2	100	50
Chr12:110913	110913771-110913747	CAAAA	5	100	50
Chr12:110906	110906346-110906309	CCT	12.3	88	58
Chr12:110891	110891354-110891312	TTTG	10.8	90	70
Chr12:110888	110888861-110888780	GAAG	20.5	97	155
Chr12:110883	110883658-110883609	CAAA	13	87	77
Chr12:110871	110871566-110871485	AAGG	20.2	81	69
Chr12:110858	110858183-110858111	ATTTT	14.4	91	121
Chr12:110831	110831568-110831534	AAC	11.7	93	61
Chr12:110828	110828354-110828186	AAAG	40	89	157
Chr12:110828	110828326-110828150	AAAGA	37.6	87	195
Chr12:110821	110821090-110821039	AAG	17	80	59
Chr12:110815	110815156-110815132	CAAAA	5	100	50
Chr12:110811	110811441-110811386	AC	28	92	94
Chr12:110805	110805983-110805957	AAAG	6.8	100	54
Chr12:110793	110793320-110793289	AT	16.5	93	57
Chr12:110793	110793289-110793259	AG	15.5	100	62
Chr12:110776	110776337-110776284	TTTC	12.8	82	54
Chr12:110776	110776182-110776129	TTTC	13	88	63
Chr12:110773	110773315-110773263	TG	26.5	100	106
Chr12:110766	110766989-110766965	ATAC	6.2	100	50
Chr12:110732	110732436-110732396	TTTCT	8.6	91	59

Chr12:110709	110709976-110709933	TTA	14.7	100	88
Chr12:110701	110701922-110701860	TGT	22.3	84	89
Chr12:110695	110695402-110695372	TG	15.5	100	62
Chr12:110666	110666453-110666423	TCTTT	6.2	100	62
Chr12:110639	110639472-110639439	CA	17	93	59
Chr12:110593.1	110593515-110593466	AT	25	91	82
Chr12:110593.2	110593135-110593098	TATT	9.5	100	76
Chr12:110572	110572896-110572870	AAAT	6.8	100	54
Chr12:110519	110519448-110519410	CAA	13	100	78
**Chr12:110517**	**110517790-110517752**	**TG**	**19.5**	**100**	**78**
Chr12:110514	110514083-110514028	AAGG	14	92	94
Chr12:110513	110513738-110513693	AAT	15.3	100	92
Chr12:110492	110492645-110492595	AATA	12.5	95	93
Chr12:110436	110436754-110436712	TG	21.5	100	86
Chr12:110430	110430443-110430402	AC	21	95	75
Chr12:110420	110420646-110420606	AAAT	10.8	89	68
Chr12:110414	110414718-110414655	AT	32	96	83
Chr12:110387	110387432-110387215	TC	109	89	339
Chr12:110384	110384248-110384206	ATTTT	8.4	82	50
Chr12:110382	110382500-110382462	TA	19.5	94	69
Chr12:110364	110364608-110364571	ATTT	9.5	100	76
Chr12:110303	110303429-110303403	AAAC	6.8	100	54
**Chr12:110302**	**110302615-110302572**	**AAT**	**14.7**	**85**	**61**
Chr12:110300	110300147-110300117	GGAGG	6.2	100	62
Chr12:110296	110296131-110296103	AAAAC	6	92	51
Chr12:110293	110293865-110293827	AC	19.5	100	78
Chr12:110293	110293412-110293386	AAAC	6.8	100	54
Chr12:110274	110274759-110274713	TTTTG	10	86	73
Chr12:110265	110265665-110265625	TTTA	10.2	100	82
Chr12:110260	110260602-110260576	TA	13.5	100	54
Chr12:110256	110256385-110256351	AC	17.5	100	70
Chr12:110240	110240667-110240641	AAAT	6.8	100	54
**Chr12:110228**	**110228537-110228488**	**AAAT**	**11.8**	**95**	**73**
Chr12:110216	110216205-110216174	AAAAC	6.4	100	64
Chr12:110201	110201626-110201600	AAAT	6.8	100	54
Chr12:110197	110197584-110197556	TA	14.5	100	58
Chr12:110197	110197293-110197201	ATCT	23.2	84	100
Chr12:110189	110189363-110189337	AAAT	6.8	100	54
Chr12:110186	110186276-110186239	AAAC	9.5	100	76
Chr12:110179	110179806-110179774	TG	16.5	100	66
Chr12:110179	110179774-110179725	TA	25	100	100
Chr12:110177	110177300-110177268	AAACA	6.6	100	66
Chr12:110169	110169082-110169014	AGGA	17.2	96	129
Chr12:110145	110145827-110145799	AAAAC	5.8	100	58
Chr12:110144	110144220-110144184	AAC	12.7	88	58
Chr12:110139	110139907-110139801	TTTTA	21.4	92	182

Chr12:110137	110137767-110137741	TTTA	6.8	100	54
**Chr12:110121**	**110121331-110121275**	**TG**	**28.5**	**96**	**105**
Chr12:110100	110100135-110100097	TTTCT	8.6	94	50
Chr12:110093	110093190-110093119	TC	36	85	99
Chr12:110092	110092672-110092629	TCCA	11	83	54
Chr12:110087	110087536-110087509	GTTT	7	100	56
Chr12:110084	110084511-110084464	TTCC	11.8	86	69
Chr12:110071	110071704-110071666	TGA	13	94	69
Chr12:110068	110068331-110068209	CTT	41	93	212
Chr12:110063	110063943-110063908	GTTT	9	93	63
Chr12:110063	110063644-110063603	AATA	10.5	100	84
Chr12:110019	110019798-110019770	AAAC	7.2	100	58
Chr12:109987	109987424-109987386	TATT	10.2	89	64
Chr12:109986	109986793-109986767	TTTG	6.8	100	54
Chr12:109980	109980410-109980374	AAAT	9.8	88	60
Chr12:109976	109976138-109976106	TG	16.5	93	57
Chr12:109966	109966683-109966649	TTTTG	7	90	52
**D12S161**	**109964676-109964621**	**GT**	**28**	**100**	**112**

[a]*Established markers are named as published. Novel markers are named with Chr12 prefix followed by their distance (in* 10^3 *base pairs) from chromosome 12pter, based on the UCSC genome browser reference sequence (GRCh38/hg38).*

[b]*Base pairs from chromosome 12pter, based on GRCh38/hg38.*

[c]*Overall percentage of matches between adjacent copies as calculated by TRF (http://tandem.bu.edu/trf/trf.html).*

[d]*Alignment score as calculated by TRF with settings as +2 for a base pair match and -7 for a mismatch or an indel in the repeat stretch. Alignment score is the weight for match, mismatch and indels.*

Black-shaded marker is the ATXN2 CAG repeat; bolded markers were included in the final heptadecaplex panel; gray-shaded markers were excluded after preliminary screening due to low PIC values, poor amplification results, or poor peak pattern and difficulty in allele calling.

Table S2. Allele frequency distribution of the 17 microsatellite markers flanking *ATXN2* $(CAG)_n$

Allele No.	Chr12:113048			Chr12:112791			Chr12:112765			Chr12:112694		
	Allele	Frequency		Allele	Frequency		Allele	Frequency		Allele	Frequency	
		CH	CAU		CH	CAU		CH	CAU		CH	CAU
1	180	0.55	0.35	207	0.02	0.00	395	0.01	0.00	250	0.01	0.00
2	182	0.04	0.03	217	0.01	0.00	397	0.00	0.01	252	0.00	0.01
3	184	0.32	0.18	219	0.00	0.05	399	0.73	0.28	254	0.00	0.01
4	186	0.09	0.22	221	0.14	0.03	401	0.12	0.19	256	0.00	0.00
5	188	0.00	0.08	223	0.03	0.01	403	0.12	0.33	258	0.01	0.04
6	190	0.00	0.05	225	0.00	0.01	405	0.03	0.15	260	0.31	0.30
7	192	0.01	0.06	227	0.01	0.01	407	0.00	0.01	262	0.05	0.01
8	194	0.00	0.01	229	0.01	0.01	409	0.00	0.03	264	0.28	0.01
9	196	0.00	0.03	231	0.01	0.01				266	0.18	0.02
10	200	0.00	0.01	233	0.02	0.01				268	0.01	0.02
11				235	0.01	0.00				270	0.07	0.54
12				237	0.13	0.01				272	0.07	0.05
13				239	0.03	0.00				274	0.01	0.02
14				241	0.04	0.06				276	0.00	0.01
15				243	0.02	0.04						
16				245	0.02	0.07						
17				247	0.03	0.06						
18				249	0.05	0.07						
19				251	0.06	0.09						
20				253	0.08	0.13						
21				255	0.07	0.08						
22				257	0.04	0.08						
23				259	0.05	0.05						
24				261	0.03	0.06						
25				263	0.02	0.03						
26				265	0.04	0.01						
27				267	0.02	0.01						
28				269	0.03	0.01						
29				271	0.00	0.01						
30				273	0.01	0.00						
31				275	0.00	0.01						
32				277	0.00	0.01						
33				279	0.00	0.01						
34				281	0.00	0.01						
35				283	0.01	0.00						
H_e		0.59	0.78		0.93	0.93		0.44	0.75		0.78	0.62
H_o		0.54	0.80		0.97	0.98		0.44	0.80		0.83	0.60

Allele No.	Chr12:112667			Chr12:111939			Chr12:111935			AFMA154TC5		
	Allele	Frequency		Allele	Frequency		Allele	Frequency		Allele	Frequency	
		CH	CAU		CH	CAU		CH	CAU		CH	CAU
1	147	0.01596	0.03	175	0.68	0.00	306	0.00	0.01	166	0.01	0.00
2	151	0.01	0.01	197	0.00	0.01	310	0.00	0.02	170	0.22	0.07
3	155	0.01	0.02	199	0.00	0.03	314	0.01	0.02	172	0.01	0.12
4	157	0.02	0.05	201	0.12	0.15	316	0.01	0.02	174	0.00	0.01
5	159	0.05	0.05	203	0.09	0.09	318	0.06	0.19	180	0.02	0.42
6	161	0.12	0.06	205	0.02	0.10	320	0.09	0.49	182	0.01	0.05
7	163	0.21	0.10	207	0.03	0.22	322	0.06	0.08	184	0.64	0.27
8	165	0.37	0.10	209	0.01	0.19	324	0.13	0.07	186	0.11	0.03
9	167	0.11	0.28	211	0.04	0.11	326	0.23	0.09	188	0.00	0.02
10	169	0.07	0.13	213	0.01	0.05	328	0.04	0.02	190	0.00	0.02
11	171	0.01	0.08	215	0.01	0.02	330	0.04	0.01			
12	173	0.01	0.07	217	0.00	0.01	332	0.03	0.01			
13	175	0.00	0.01	221	0.00	0.01	334	0.06	0.00			
14	177	0.00	0.01	223	0.00	0.01	336	0.06	0.00			
15							338	0.14	0.00			
16							340	0.03	0.00			
17							342	0.01	0.00			
H_e		0.78	0.86		0.51	0.85		0.88	0.70		0.53	0.73
H_o		0.80	0.91		0.54	0.88		0.88	0.72		0.54	0.73

Allele No.	Chr12:111483			Chr12:111464			Chr12:111380			Chr12:111336		
	Allele	Frequency		Allele	Frequency		Allele	Frequency		Allele	Frequency	
		CH	CAU		CH	CAU		CH	CAU		CH	CAU
1	414	0.00	0.02	411	0.00	0.01	360	0	0.01	361	0.00	0.01
2	426	0.00	0.01	413	0.01	0.01	362	0.10106	0.75	373	0.01	0.00
3	428	0.00	0.02	415	0.04	0.01	364	0.58	0.04	375	0.73	0.17
4	432	0.04	0.00	417	0.04	0.64	366	0.04	0.01	377	0.02	0.01
5	434	0.03	0.00	419	0.51	0.08	368	0.00	0.02	379	0.01	0.00
6	436	0.52	0.00	421	0.17	0.06	370	0.20	0.14	383	0.03	0.72
7	438	0.07	0.05	423	0.20	0.11	372	0.07	0.03	385	0.01	0.05
8	440	0.13	0.06	425	0.04	0.08	374	0.01	0.01	387	0.00	0.01
9	442	0.14	0.61	427	0.00	0.01	376	0.01	0.00	389	0.17	0.04
10	444	0.06	0.17							391	0.03	0.01
11	446	0.01	0.05							393	0.01	0.00
12	448	0.01	0.01									
13	450	0.01	0.00									
H_e		0.68	0.58		0.67	0.56		0.61	0.41		0.43	0.45
H_o		0.69	0.54		0.74	0.60		0.61	0.41		0.49	0.47

Allele No.	Chr12:110517			Chr12:110302			Chr12:110228			Chr12:110121		
	Allele	Frequency		Allele	Frequency		Allele	Frequency		Allele	Frequency	
		CH	CAU		CH	CAU		CH	CAU		CH	CAU
1	194	0.03	0.00	441	0.08	0.14	285	0.01	0.02	309	0	0.01
2	202	0.05	0.00	444	0.66	0.35	289	0.91	0.69	311	0.02	0.12
3	204	0.03	0.09	447	0.26	0.43	293	0.07	0.10	313	0.04	0.15
4	206	0.11	0.06	450	0.00	0.07	297	0.01	0.03	315	0.00	0.10
5	208	0.07	0.17	453	0.00	0.01	301	0.01	0.13	317	0.44	0.33
6	210	0.48	0.41				305	0.00	0.03	319	0.05	0.03
7	212	0.19	0.26				309	0.00	0.01	321	0.25	0.08
8	214	0.04	0.01							323	0.15	0.15
9	216	0.01	0.01							325	0.04	0.03
10										327	0.01	0.01
H_e		0.71	0.73		0.49	0.66		0.16	0.50		0.72	0.81
H_o		0.70	0.77		0.51	0.77		0.17	0.56		0.65	0.86

Allele No.	D12S161		
	Allele	Frequency	
		CH	CAU
1	232	0.00	0.12
2	236	0.00	0.01
3	240	0.00	0.01
4	242	0.01	0.01
5	244	0.04	0.02
6	246	0.05	0.06
7	248	0.10	0.06
8	250	0.08	0.08
9	252	0.19	0.14
10	254	0.14	0.15
11	256	0.11	0.13
12	258	0.10	0.08
13	260	0.10	0.07
14	262	0.05	0.04
15	264	0.01	0.01
16	266	0.01	0.01
17	268	0.00	0.01
18	270	0.01	0.00
19	272	0.01	0.00
H_e		0.89	0.90
H_o		0.89	0.89

Table S3. SCA2 heptadecaplex microsatellite marker genotypes from 5-cell WGA products of 3 different cell lines.

5-cell WGA Samples	Chr12:113048		Chr12:112791		Chr12:112765		Chr12:112694		Chr12:112667		Chr12:111939		Chr12:111935		AFMA154TC5	
GM17942-MC1	186	202	241	245	399	401	262	270	159	167	203	205	320	324	178	184
GM17942-MC2	186	202	241	245	399	401	262	270	159	167	203	205	320	324	178	184
GM17942-MC3	186	202	241	245	399	401	262	270	159	167	203	205	320	324	178	184
GM22601-MC1	180	192	247	265	401	405	ADO	270	157	159	203	213	322	324	184	184
GM22601-MC2	180	192	247	265	401	405	260	270	157	159	203	213	322	324	184	184
GM22601-MC3	180	192	247	265	401	405	260	270	157	159	203	213	322	324	184	184
GM50194-MC1	190	196	245	249	401	403	260	274	147	159	199	203	320	322	180	184
GM50194-MC2	190	196	245	249	401	403	260	274	147	159	199	203	320	322	180	184
GM50194-MC3	190	196	245	249	401	403	260	274	147	159	199	203	320	322	180	184

5-cell WGA Samples	Chr12:111483		Chr12:111464		Chr12:111380		Chr12:111336		Chr12:110517		Chr12:110302		Chr12:110228		Chr12:110121		D12S161	
GM17942-MC1	438	444	401	421	372	372	375	375	204	208	441	447	293	305	307	313	ADO	260
GM17942-MC2	438	444	401	421	372	372	375	375	204	208	441	447	293	305	307	313	250	260
GM17942-MC3	438	444	401	421	372	372	375	375	204	208	441	447	293	305	307	313	250	260
GM22601-MC1	440	446	423	431	362	374	375	393	204	214	432	450	289	289	323	323	250	254
GM22601-MC2	440	446	423	431	362	374	375	393	204	214	432	450	289	289	323	323	250	254
GM22601-MC3	440	446	423	431	362	374	375	393	204	214	432	450	289	289	323	323	250	254
GM50194-MC1	442	446	417	421	362	362	383	383	206	210	444	450	289	289	317	323	254	256
GM50194-MC2	442	446	417	421	362	362	383	383	206	210	444	450	289	289	317	323	254	256
GM50194-MC3	442	446	417	421	362	362	383	383	206	210	444	450	289	289	317	323	254	256
